# MRI-predicted extramural vascular invasion and tumour deposit are main predictors of disease-free survival in patients undergoing surgical resection for rectal cancer

**DOI:** 10.1093/bjsopen/zrad139

**Published:** 2024-01-03

**Authors:** Francesco Di Fabio, Niccolo Allievi, Amy Lord, Anisha Bhagwanani, Arcot Venkatasubramaniam, Steve Arnold, Brendan Moran

**Affiliations:** Rectal Cancer Unit, Colorectal Surgery, Basingstoke and North Hampshire Hospital, Basingstoke, UK; Rectal Cancer Unit, Colorectal Surgery, Basingstoke and North Hampshire Hospital, Basingstoke, UK; Rectal Cancer Unit, Colorectal Surgery, Basingstoke and North Hampshire Hospital, Basingstoke, UK; Radiology, Basingstoke and North Hampshire Hospital, Basingstoke, UK; Rectal Cancer Unit, Colorectal Surgery, Basingstoke and North Hampshire Hospital, Basingstoke, UK; Rectal Cancer Unit, Colorectal Surgery, Basingstoke and North Hampshire Hospital, Basingstoke, UK; Rectal Cancer Unit, Colorectal Surgery, Basingstoke and North Hampshire Hospital, Basingstoke, UK

## Abstract

**Background:**

MRI is crucial in staging patients with rectal cancer and planning treatment. The aim was to analyse the prognostic role of MRI-predicted tumour deposits and/or extramural vascular invasion (mrTD/EMVI) in a cohort of patients with rectal cancer undergoing surgical resection, with selective neoadjuvant chemoradiotherapy (nCRT).

**Method:**

Retrospective analysis of a single-centre cohort of consecutive patients with rectal cancer undergoing low anterior resection or abdominoperineal excision between 2008 and 2020. Unit policy was selective nCRT for MRI-predicted threatened or involved circumferential resection margin (mrCRM), or radiologically involved pelvic sidewall nodes. The primary outcome was disease-free survival. Secondary outcomes were rates of local recurrence, distant recurrence and overall survival.

**Results:**

A total of 314 patients were analysed. Median age was 65 years (female/male: 114/200). A total of 54/314 (17%) had nCRT and 35 patients (11%) underwent abdominoperineal excision. Median follow-up was 64 months. Overall, local recurrence was detected in 18/314 (5.7%) and distant recurrence in 45/314 (14.3%). In patients not receiving nCRT (*n* = 260), local recurrence was detected in 11/260 (4.2%) and distant recurrence in 35/260 (13.5%). Disease-free survival was 80.5% at 5 years. Specifically, disease-free survival was 89% in mrTD/EMVI-negative and mrCRM-negative, 67% in mrTD/EMVI-positive and mrCRM-negative, and 64% in the mrCRM-positive rectal cancer (log-rank, *P* < 0.001). On multivariable Cox-regression analysis mrTD/EMVI was the only MRI variable associated with disease-free survival (hazard ratio 2.95; *P* < 0.001).

**Conclusion:**

mrTD/EMVI is a major prognostic indicator. Rectal cancer patients with mrCRM-negative and mrTD/EMVI-negative have excellent long-term outcomes with surgery alone. Patients with mrTD/EMVI-positive should be selectively stratified for neoadjuvant treatments in future clinical trials.

## Teaser

mrTD/EMVI is a major prognostic indicator in rectal cancer. A risk stratification for recurrence disease based on the presence of mrTD and mrEMVI outperforms the ‘classical’ stratification based solely on the MRI-predicted TNM status.

## Introduction

The management of rectal cancer is complex and rapidly evolving. The crucial components of rectal cancer treatment are optimal decision-making, precision surgery when needed, selective use of neoadjuvant and adjuvant treatment, and patients’ preference. MRI is pivotal in the decision-making process for local staging of rectal cancer and is key in establishing treatment strategies such as neoadjuvant therapy or selection of patients who can go straight to surgery.

Decisions regarding management of rectal cancer are commonly based on CT- and MRI-predicted TNM classification. MRI is most accurate in assessing extramural depth of invasion and relationship of the tumour to the mesorectal fascia (the MRI-predicted circumferential resection margin, or mr-CRM). However, sensitivity and specificity of MRI in predicting lymph node metastases is suboptimal^1,2^, with a tendency to overstage the mesorectal nodal status.

It is important to note that there is increasing evidence that MRI-based risk stratification is not limited to the classical pathological TNM variables^3^. Specifically, MRI-predicted extra-mural vascular invasion (mrEMVI), and tumour deposit (mrTD) seem to have a major prognostic significance, as recently emerging in clinical studies^3^. MRI provides a three-dimensional visualization of the mesorectum that can maximize assessment of EMVI and TD. Hypothetically, EMVI and TD might be more likely to be missed at histopathological evaluation, especially if 1-cm thick pathological slices are used to sample the mesorectum. Reports suggest that mrEMVI is detected on MRI with a sensitivity and specificity of 100% and 89%, respectively, and mrEMVI has been shown to be associated with poorer survival outcomes, with increased rates of distant recurrence^4,5^. Likewise, mrTD represents extensive manifestations of extramural venous and perineural invasion, with negative prognostic implications related to increased risk of distant metastases and poorer disease-free and overall survival (OS)^6^. It appears that mrTD is comparable to mrEMVI in characterizing a broader ‘vascular invasion pathway’ leading to haematogenous distant metastases. This hypothesis is supported by the significant association of TD with EMVI in a recent meta-analysis of publications on over 19 000 patients^6^.

The study reports on a cohort of patients with rectal cancer undergoing surgical resection where selective neoadjuvant chemoradiotherapy (nCRT) is the unit policy in case of MRI-predicted margin involvement or MRI-predicted pelvic sidewall nodal involvement. Based on previous publications and ongoing debate suggesting that tumour deposits and extramural vascular invasion may be similar, both were combined into the category ‘mrTD/EMVI’.

The primary objective of the current study was to analyse the prognostic role of MRI-predicted tumour deposit and/or MRI-predicted extra mural vascular invasion (mrTD/EMVI) on disease-free survival (DFS).

## Patients and methods

This is a retrospective observational single-centre study on prospectively collected data of consecutive patients with rectal cancer undergoing low anterior resection (LAR) or abdominoperineal excision (APE) between 2008 and 2020. The data are based on service delivery and assessment and do not require formal ethics committee approval. This study was conducted according to the STROBE guidelines.

Patients with a diagnosis of primary rectal cancer undergoing LAR or APE, with or without nCRT, in Basingstoke North Hampshire Hospital (UK), Tertiary Referral Centre, between July 2008 and July 2020 were included. Patients with rectal cancer were discussed in a dedicated colorectal cancer multidisciplinary team (MDT) meeting, where treatment was planned.

A rectal cancer was arbitrarily defined as a tumour with its origin within 15 cm of the anal verge, measured with a rigid sigmoidoscopy in an awake patient in the left lateral position. Low rectal cancer was more objectively defined as a tumour with its lower edge at or below the origins of the levator on the pelvic sidewall, as defined in the ‘LOREC’ national programme. This is usually within 6 cm of the anal verge^7^.

The MDT and unit policy was to consider nCRT for MRI-predicted threatened or involved mesorectal fascia (mrCRM-positive), radiologically predicted involved pelvic sidewall nodes, or a low rectal cancer with involvement of the inter-sphincteric space or external sphincter. Patients who underwent nCRT had 45–50.4 Gy in 25 or 28 fractions, combined with oral capecitabine or 5-fluorouracil. From 2010 induction chemotherapy before CRT was routinely adopted (four cycles of FOLFOX). The planned interval between the end of nCRT and surgery was 12 weeks. Two patients had neoadjuvant chemotherapy only and were included in the nCRT group.

Exclusion criteria were age < 18 years, early rectal cancer excised by local excision only, distant metastases at presentation, rectal cancer with concomitant peritoneal disease treated by cytoreductive surgery and Hyperthermic Intraoperative Chemotherapy (HIPEC), locally advanced rectal cancer requiring complete pelvic exenteration or where staging pelvic MRI was not available. Patients with a complete clinical response (cCR) after nCRT who were on the Watch & Wait–deferral of surgery (W&W) pathway were also excluded. Demographic data, clinical variables, radiological data, pathology results and follow-up data were obtained from an electronic database and hospital records.

Prognostic MRI variables analysed were MRI-predicted T stage (mrT), MRI nodal staging (mrN), mrCRM, mrTD and mrEMVI. The mrT stage was dichotomized as mrT1–2 *versus* mrT3–4. The mrCRM was defined as ‘positive’ if the distance from the primary tumour, MRI-predicted positive nodes or mrTD/EMVI to the mesorectal fascia was less than 1 mm^8^. The definition of mrTD on MRI was irregular nodules within the mesorectum that directly interrupt the course of veins but are discontinuous from the primary tumour. Tumour deposits can be distinguished from lymph node metastases as they cannot be separated from the vein when assessed on two orthogonal views and tend to taper into the vein (comet-tail appearance) rather than being alongside the vein and forming an acute angle, while mrEMVI is defined as a contiguous expansion of perirectal veins with intermediate tumour signal intensity. We amalgamated mrTD and mrEMVI into mrTD/EMVI, as these two features represent two signs of a broader ‘vascular invasion pathway’ and have previously been shown to have a similar prognostic impact on distant failure^9,10^. In this context, mrTD/EMVI was considered positive when one, or both, of the variables were detected. There is no specific sign-off for competency in reporting rectal MRI in the UK. Rectal MRI scans were reported by dedicated lower gastrointestinal tract radiologists who had 2–3 years of subspecialty training and experience in reporting rectal MRI scans as part of a rectal cancer MDT meeting. For the purpose of this study, all MRI scans were re-reviewed by an experienced gastrointestinal radiologist (A.B.) in a blinded setting.

Patients with rectal cancer were stratified into three prognostic groups based on MRI features: low risk (mrTD/EMVI-negative, mrCRM-clear), moderate risk (mrTD/EMVI-positive, mrCRM-clear) or high risk (any mrTD/EMVI, mrCRM-positive).

The primary outcome was DFS. Secondary outcomes were rates of local recurrence (LR) and distant recurrence (DR) and OS. The follow-up period was calculated from the date of surgery to last censor and minimum follow-up was 2 years. DFS was defined as the number of patients alive and free from local or distant cancer recurrence at the date of censor. OS was defined as the number of patients alive at the date of censor. LR was defined as any recurrence in the pelvis. Recurrent disease outside the pelvis was defined as DR.

### Statistical analysis

Baseline characteristics and unadjusted outcomes were compared using the Kruskal–Wallis or the Student’s *t*-test for continuous variables and the Pearson χ^2^ test for categorical variables. Binomial logistic regression was used to study the relationship between baseline and relevant clinical characteristics with the incidence of DR; the Haldane–Ascomb correction was used in case a zero-cell count was encountered in 2 × 2 tables. Survival was determined using the Kaplan–Meier method. The Log-Rank test was used to assess differences between survival curves. Cox proportional hazard regression model was used for multivariable analysis of prognostic MRI variables in relation to DFS and OS. In multivariable analysis, mrEMVI and mrTD were combined due to multicollinearity (Spearman test; *P* = 0.01).

Data analyses were performed using the SPSS Software (version 20.0; IBM Corp, Armonk, New York) and R software (version 3.5.3; R Core Team 2019, Vienna, Austria). All *P* were two-sided and were considered significant when <0.05.

## Results

A total of 314 patients with rectal cancer who underwent LAR or APE in the study period were included. The median age was 65 years and 114/314 (36%) were females. Overall, 54/314 (17%) had nCRT. In total, 279 (89%) had restorative LAR with 35 (11%) patients having APE.

*Table 1* summarizes the demographic, radiological and pathological staging data in the group not receiving neoadjuvant treatment (non-nCRT) compared with the group of patients who underwent nCRT. Overall, 38/314 (12%) patients were mrCRM-positive and 25/38 (66%) were mrTD/EMVI-positive.

**Table 1:** Demographic, radiological (MRI) and pathological staging data

Patient characteristics (*n* = 314)	Overall	Preop. CRT not received (*n* = 260)	Preop. CRT received (*n* = 54)	*P*
Age at diagnosis (years), median (i.q.r.)	65 (57–71)	65 (57–72)	65 (54–71)	0.407
**Sex**				
Male	200 (63.7)	159 (61.8)	41 (75.9)	0.040
Female	114 (36.3)	101 (38.8)	13 (24.1)	
**mrT stage**				
1–2	127 (40.4)	124 (47.7)	3 (5.6)	<0.001
3–4	187 (59.6)	136 (52.3)	51 (94.4)	
mrN pos	133 (42.4)	94 (36.2)	39 (72.2)	<0.001
mrTD	53 (16.9)	37 (14.2)	16 (29.6)	0.006
mrEMVI	97 (30.9)	65 (25)	32 (59.3)	<0.001
mrTD/EMVI	109 (34.7)	75 (28.8)	34 (63)	<0.001
mrCRM pos	38 (12.1)	11 (4.2)	27 (50)	<0.001
**MRI prognostic groups**				
Low-risk (CRM– TD/EMVI−)	192 (61.1)	178 (68.5)	14 (25.9)	<0.001
Moderate-risk (CRM– TD/EMVI+)	84 (26.8)	71 (27.3)	13 (24.1)	
High-risk (CRM+)	38 (12.1)	11 (4.2)	27 (50)	
Distance from anal verge (cm), median (i.q.r.)	8 (5.4–11)	8.2 (6.0–11.0)	6 (3.7–8.0)	<0.001
Distance from anal verge ≤6 cm	104 (33.1)	75 (28.8)	29 (53.7)	<0.001
**Operation**				
Low anterior resection	279 (88.9)	249 (95.8)	30 (55.6)	<0.001
Abdominoperineal excision	35 (11.1)	11 (4.2)	24 (44.4)	
Laparoscopic resection	161 (51.3)	144 (55.4)	17 (31.5)	0.001
**pT stage**				
0	5 (1.6)	1 (0.4)	4 (7.4)	<0.001
1–2	144 (45.9)	126 (48.5)	18 (33.3)	
3–4	165 (52.5)	133 (51.1)	32 (59.3)	
**Tumour differentiation (*n* = 306)**				
Well/moderately	291 (92.7)	244 (95.7)	47 (92.2)	0.287
Poorly	15 (4.8)	11 (4.3)	4 (7.8)	
pN pos	116 (36.9)	96 (36.9)	20 (37)	0.987
pEMVI	74 (23.8)	59 (22.9)	15 (28.3)	0.397
pTD	6 (2.1)	5 (2.1)	1 (2)	0.976
pTD/EMVI	75 (23.9)	60 (23.1)	15 (27.8)	0.461
pR 1/2 (CRM)	10 (3.2)	6 (2.3)	4 (7.4)	0.090
Adjuvant treatment received (*n* = 295)	114 (38.6)	93 (37.7)	21 (43.8)	0.427

Values are *n* (%) unless otherwise indicated. mr, MRI-predicted; p, pathological; EMVI, extramural vascular invasion; TD, tumour deposit; CRM, circumferential resection margin; CRT, chemoradiotherapy.

A total of 11 patients in the non-nCRT group had mrCRM involved but did not undergo nCRT for the following reasons: in five patients who had upper rectal cancer involving the peritoneal reflection and in one patient who developed a metachronous low rectal cancer after sigmoid resection 20 years earlier, nCRT was deemed unsafe due to risk of radiation enteritis; one patient presented with a low rectal cancer involving the external sphincter during the COVID-19 pandemic in 2020 and nCRT was not performed to minimize COVID-19-related risks; in one patient a suspicious lymph node was seen <1 mm from the mrCRM. Three patients opted not to undergo nCRT due to concerns related to risks of side effects and negative consequences on quality of life. In this subgroup, 1/11 (9%) of patients had pathological CRM involvement.

Overall, 27 patients in the nCRT group had mrCRM-predicted clear margins but had nCRT due to the presence of mrTD/EMVI in 13 or a low rectal cancer location <6 cm from the anal verge in 15, and in nine patients where both features were present.

The CRM was positive in 10 patients (3.2%) at pathology of the resected specimen. There was no pathologically involved distal resection margin (*Table 1*).

The median follow-up was 64 months. In this period, LR was detected in 18/314 (5.7%) and DR in 45/314 (14.3%). In patients not receiving nCRT (*n* = 260), LR was detected in 11/260 (4.2%) and DR in 35/260 (13.5%) (*Table 2*).

**Table 2:** Characteristics of disease recurrence

Patient characteristics (*n* = 314)	Overall	Preop. CRT not received (*n* = 260)	Preop. CRT received (*n* = 54)	*P*
Disease recurrence	56 (17.8)	40 (15.4)	16 (29.6)	0.013
Local recurrence	18 (5.7)	11 (4.2)	7 (13)	0.012
Distant recurrence	45 (14.3)	35 (13.5)	10 (18.5)	0.485
**Recurrence location**				
Local recurrence	18 (5.7)	11 (4.2)	7 (12.9)	0.331
Liver	17 (5.4)	15 (5.7)	2 (3.7)	
Lung	30 (9.5)	23 (8.8)	7 (12.9)	
Peritoneum	4 (1.2)	4 (1.5)	0	
Bone	1 (0.03)	1 (0.04)	0	
Kidney	1 (0.03)	0	1 (1.8)	
**Type of recurrence**				
Local only	11 (3.5)	5 (1.9)	6 (11.1)	0.020
Distant only	38 (12.1)	29 (11.1)	9 (16.6)	
Local + distant	7 (2.2)	6 (2.3)	1 (1.9)	

Values are *n* (%) unless otherwise indicated. CRT, chemoradiotherapy.

**Fig. 1:**
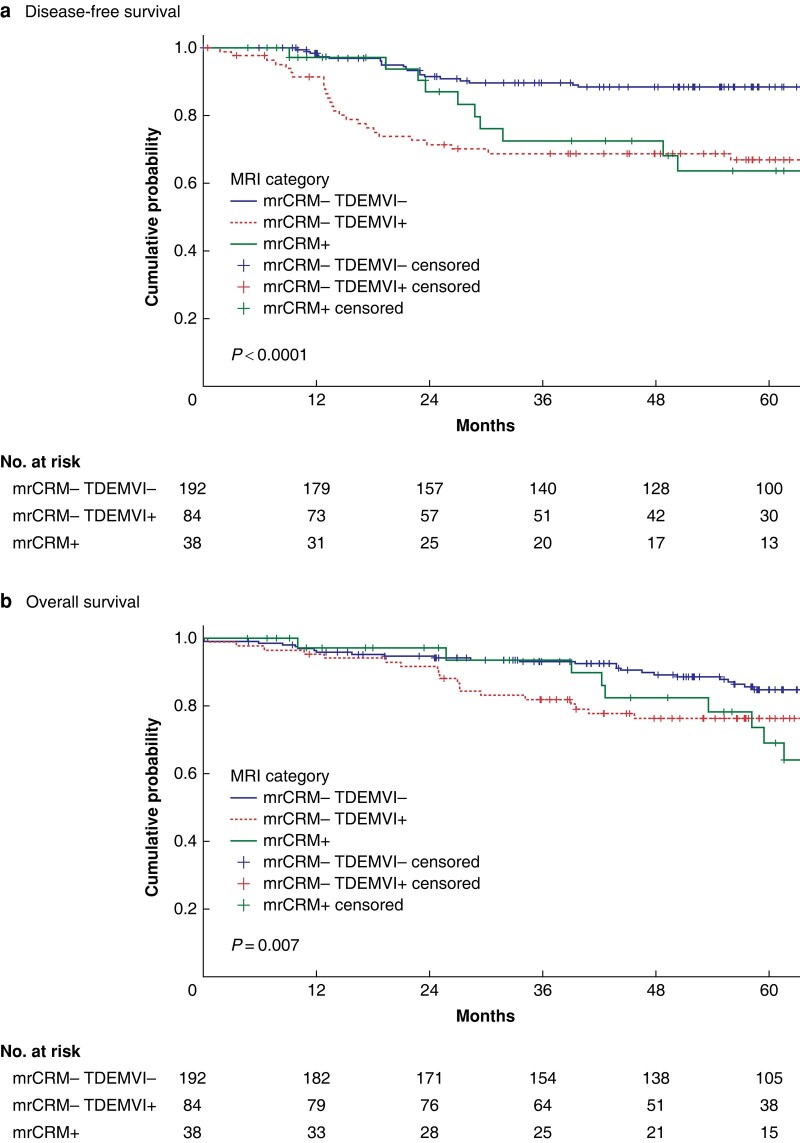
**a** Disease-free and **b** overall survival of patients with rectal cancer treated with surgical resection by MRI risk groups: low-risk (mrEMVI-negative, mrTD-negative, mrCRM-clear), moderate-risk (mrEMVI-positive or/and mrTD-positive, mrCRM-clear) or high-risk (mrCRM-positive). mr, MRI-predicted; EMVI, extramural vascular invasion; TD, tumour deposit; CRM, circumferential resection margin.

Overall, DFS was 82.2% at 3 years and 80.5% at 5 years, while OS was 89.9% at 3 years and 84.4% at 5 years. Patients in the ‘low-risk’ MRI prognostic group had better survival (DFS 89%, OS 84.8% at 5 years), as compared to the ‘moderate-risk’ and ‘high-risk’ groups (DFS 66.8% and 63.7%, OS 76.2% and 68.9% at 5 years, respectively, log-rank *P* < 0.001 for DFS and *P* = 0.007 for OS; *Fig. 1*).

In patients who had postoperative adjuvant chemotherapy, 5-year DFS was 64.4% *versus* 91.2% in those who did not have adjuvant therapy (*P* < 0.001, log-rank), and 5-year OS was 78.1% *versus* 86.7% (*P* = 0.130, log-rank).

Multivariable analysis of MRI prognostic factors showed that mrTD/EMVI was the only variable significantly associated with worse DFS (HR 2.95) and OS (HR 1.69) (*Table 3*).

**Table 3:** Multivariable analysis of the effect of demographic and MRI variables on disease-free survival and overall survival

Patient characteristics	Disease-free survival			Overall survival		
	HR	95% c.i.	*P*	HR	95% c.i.	*P*
Age ≥ 65 years	0.99	0.97,1.01	0.866	1.44	1.15,1.66	0.002
Gender	0.95	0.55,1.65	0.398	1.04	0.45,2.44	0.180
mrT3–4	2.07	0.99,4.33	0.052	1.25	0.68,2.29	0.475
mrN+	0.89	0.51,1.54	0.666	1.54	0.92,2.59	0.104
mrTD/EMVI+	2.95	1.61,5.42	<0.001	1.69	1.01,2.84	0.049
mrCRM+	1.12	0.55,2.26	0.752	1.42	0.73,2.75	0.304

mr, MRI-predicted; EMVI, extramural vascular invasion; TD, tumour deposit; CRM, circumferential resection margin.

**Fig. 2:**
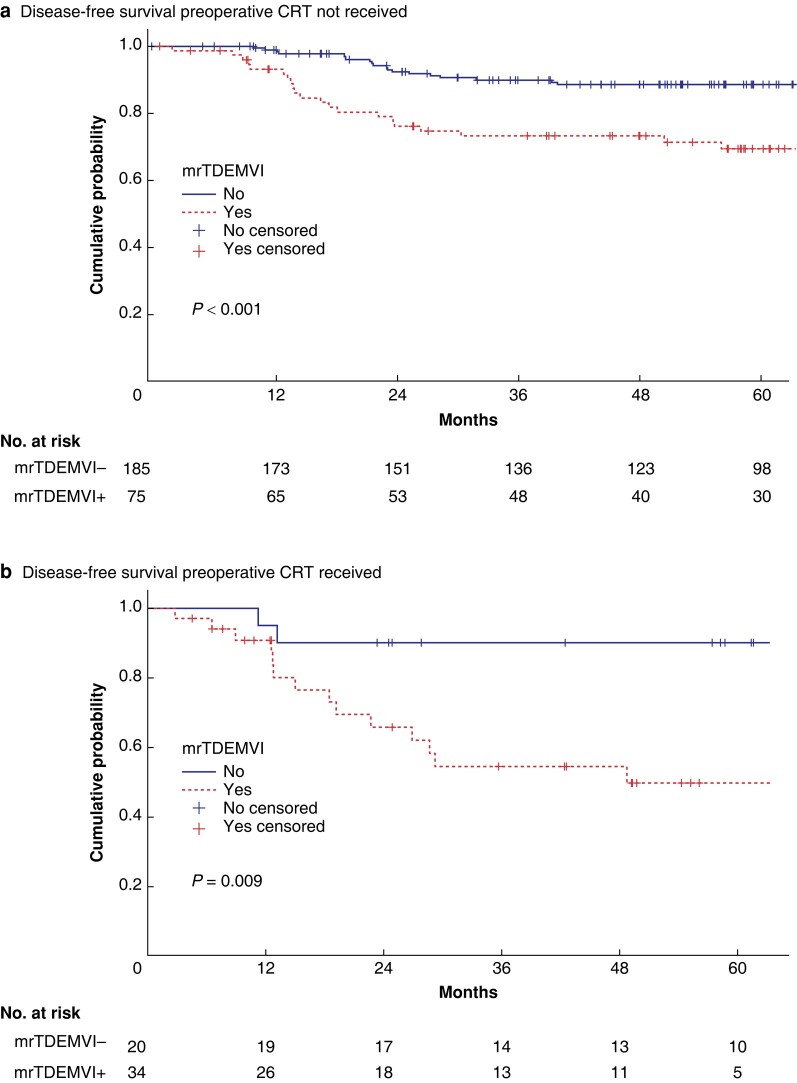
Disease-free survival of patients with rectal cancer treated with surgical resection by mrTD/EMVI status in (a) non-nCRT group and (b) nCRT group mr, MRI-predicted; EMVI, extramural vascular invasion; TD, tumour deposit; CRT, chemoradiotherapy; nCRT, neoadjuvant chemoradiotherapy.

A subgroup survival analysis was performed in non-nCRT and nCRT patients to assess the relative prognostic role of mrTD/EMVI for DFS and OS. Within the non-CRT group, 5-year DFS was 88% for patients mrTD/EMVI-negative and 69% for mrTD/EMVI-positive (log-rank, *P* < 0.001) and the 5-year OS was 85% *versus* 76% (log-rank, *P* = 0.039; *Fig. 2*). Within the nCRT group 5-year DFS was 90% for patients mrTD/EMVI-negative *versus* 49% for mrTD/EMVI-positive (log-rank, *P* = 0.009) and 5-year OS was 78% *versus* 49% (log-rank, *P* = 0.020).

Variables associated with crude LR and DR are summarized in *Table 4*. MRI prognostic factors significantly associated with crude LR were mrT3–4 status (*P* = 0.049) and associated with crude DR were mrT3–4 status (*P* = 0.010), and the presence of mrEMVI (*P* < 0.001) and mrTD (*P* < 0.001).

**Table 4:** Variables associated with crude local and distal recurrence

Variables	Local recurrence	*P*	Distant recurrence	*P*
**Sex**				
Male Female	14 (7.0) 4 (3.5)	0.201	27 (13.5) 18 (15.8)	0.578
**Age**				
<65 ≥65	9 (6.0) 8 (5.0)	0.689	25 (16.8) 18 (11.3)	0.161
**Operation**				
LAR APE	13 (4.7) 5 (14.3)	0.021	34 (12.2) 11 (24.4)	0.002
**Tumour height from anal verge**				
≤6 cm > 6 cm	12 (11.5) 6 (2.9)	0.003	19 (18.3) 26 (12.4)	0.161
**Pre-operative MRI variables**				
**mrT status**				
1–2 3–4	4 (3.1) 14 (7.5)	0.049	10 (7.9) 35 (18.7)	0.007
**mrN status**				
Negative Positive	9 (5.0) 9 (6.8)	0.499	24 (13.3) 21 (15.8)	0.527
**mrEMVI**				
Negative Positive	12 (5.5) 6 (6.2)	0.817	19 (8.8) 26 (26.8)	<0.001
**mrTD**				
Negative Positive	14 (5.4) 4 (7.5)	0.533	28 (10.7) 17 (32.1)	<0.001
**mrTD/EMVI**				
No Yes	10 (4.9) 8 (7.3)	0.372	17 (8.3) 28 (25.7)	<0.001
**mrCRM**				
Free Threatened/Involved	16 (5.8) 2 (5.3)	0.894	37 (13.4) 8 (21.1)	0.207
**Post-operative pathological variables**				
**pT status**				
1–2 3–4	5 (3.4) 13 (7.9)	0.085	12 (8.1) 33 (20.0)	0.003
**pN status**				
Negative Positive	9 (4.5) 9 (7.8)	0.237	16 (8.2) 29 (25.0)	<0.001
**pEMVI**				
Negative Positive	12 (5.1) 6 (8.1)	0.327	24 (10.0) 21 (28.4)	<0.001
**pTD**				
Negative Positive	17 (5.6) 1 (16.7)	0.252	43 (14.0) 2 (33.3)	0.194
**pTD/EMVI**				
No Yes	12 (5) 6 (8)	0.333	24 (10) 21 (28)	<0.001
**pR (CRM) status**				
R0 R1–2	17 (5.6) 1 (10.0)	0.555	42 (13.8) 3 (30.0)	0.151
**Poorly differentiated cancer**				
No Yes	16 (5.5) 2 (13.3)	0.209	38 (13.1) 7 (46.7)	<0.001

Values are *n* (%) unless otherwise stated. mr, MRI-predicted; p, pathological; EMVI, extramural vascular invasion; TD, tumour deposit; CRM, circumferential resection margin; LAR, low anterior resection; APE, abdominoperineal excision.

## Discussion

This study demonstrates that patients with rectal cancer with ‘low-risk’ MRI prognostic factors (mrCRM-clear and mrTD/EMVI-negative) have excellent long-term outcomes with optimal curative surgery alone (DFS 89% and OS 85% at 5 years). In addition, using high-risk MRI features as a selective indication for nCRT in patients with rectal cancer resulted in a low rate of involved pathological circumferential margin (pCRM) of 3.2%, a low LR of 5.7% and good survival outcomes (5-year DFS of 80.5% and OS of 84.4%). However, mrTD/EMVI appears to be the main determinant for DFS with mrTD/EMVI involvement having a hazard ratio of 2.95 for DFS at multivariable analysis (*Table 3*).

TD and EMVI are believed to represent crystallized events of a continuum constituting the ‘vascular pathway’ leading to haematogenous metastases^10^, and their role in the development of DR^4,5,11,12^. It is increasingly recognized that TD and lymph node metastases have different appearances at high-resolution pelvic MRI, although some overlap in diagnosis between the two entities exists and this is partially due to evolving definitions^13^. However, our data confirm that the presence of mrTD is associated with worse prognosis as compared to involved mrN^6,12^.

The discrepancy found between mrTD and pTD has been previously observed^3^. On MRI, mrTDs can be distinguished from involved mrNs as they cannot be separated from the vein when assessed on two orthogonal views and tend to taper into the vein (described as a comet-tail appearance), rather than being alongside the vein and forming an acute angle. This relationship with veins is not always evident on histopathology, due to limited sections and the fact that the vessels may have been compromised by radiotherapy within the area being examined. The three-dimensional MRI offers objective advantages allowing visualization of the relationship of mrTDs to venous anatomy.

In the current study, mrTD/EMVI presence or absence was the only preoperative MRI feature associated with DFS and OS at multivariable analysis. In addition, within the non-nCRT and nCRT groups, mrTD/EMVI was associated with significantly poorer outcomes. In fact, rectal cancer patients undergoing nCRT with mrCRM involved but mrTD/EMVI-negative have good long-term outcomes (*Fig. 2*).

It has previously been reported that nCRT has been shown to be effective in converting almost 50% of patients with an mrEMVI-positive status to mrEMVI-negative, with survival benefit^14,15^. Nevertheless, it is unclear which component of the chemoradiotherapy is effective against the ‘vascular pathway’. A post-hoc analysis of the GEMCAD trial^16^ has shown that 50% of patients with mrEMVI-positive status are converted to an mrEMVI-negative status (vascular responders), confirming that chemotherapy alone might partially address vascular invasion in rectal cancer.

Our data show that the rate of LR is relatively low. However, DR rates remain problematic with significant clinical events in 14% of patients and 19% in patients who had neoadjuvant therapy. The low LR among patients who did not receive preoperative CRT in this study (4.2%) confirms that optimal local control can be achieved with appropriate preoperative recognition of high-risk features for LR and selective nCRT in this group^17,18^ and high-quality surgery for all^19,20^. The careful assessment of the CRM on MRI, as reported in the MERCURY study^8^, and surgical precision of surgery using the principles of total mesorectal excision, are key features in achieving low rates of pelvic recurrence. In the MERCURY study, where preoperative MRI was correlated with pathological assessment of the resected specimen, MRI accurately predicted extramural depth of invasion to within 0.5 mm, which makes it the optimal imaging modality for assessing higher-risk mrT3 tumours^8^. In addition, MRI predicted the relationship of the tumour to the mesorectal fascia and an mrCRM clear margin was reported with an accuracy of 94% at detailed pathological assessment of the resected specimen^20^.

Substantial variation exists in the indication for neoadjuvant treatment across the UK, with on average 37% of patients with rectal cancer receiving nCRT^21^. The current indications for nCRT within the National UK NICE guidelines^22^ are controversial and seem to be associated with possible overtreatment in a proportion of patients^23^. The selective criteria adopted in our unit resulted in a relatively small proportion of patients undergoing nCRT (17%)^24^. Notably, mrCRM in multivariable analysis was not an independent risk factor for DFS, suggesting that selective nCRT and optimal precision surgery might mitigate the negative prognosis of mrCRM-involved rectal cancer. A recent national cross-sectional cohort study in the Netherlands reported that a reduction in preoperative radiotherapy use by 50% for non-locally advanced rectal cancer did not compromise cancer-related outcomes and was instead associated with improved overall survival^25^: in 2011, neoadjuvant radiotherapy was used in 86% of patients with rectal cancer, while this proportion was 37% in the 2016 cohort. The LR at 4 years was 5.8% and 5.5%, respectively (*P* = 0.999). In the 2016 cohort, 4-year OS was significantly higher (86.4% *versus* 79.6%; *P* < 0.001) with lower non-cancer-related mortality (6.7% *versus* 13.7%; *P* < 0.001).

There are several limitations in this study. This is a retrospective study of a cohort of patients treated at a single centre over a period of 12 years, with intrinsic biases including variations in clinical practice, particularly related to increased adoption of a deferral of surgery and W&W approach after a cCR. The W&W approach after cCR tends to self-select a cohort of patients with potentially better prognosis, and the decision to exclude this cohort from the study may have contributed to an overestimation of negative results. Finally, the reduced number of events, specifically related to LR, limited the multivariable analysis and some associations may have been missed.

A risk stratification for recurrence disease based on the presence of mrTD and mrEMVI outperforms the ‘classical’ stratification based solely on the MRI-predicted TNM status^3^, which is the basis of current UK NICE guidelines. Further research is needed, mrTD/EMVI should be routinely reported, and patients selectively stratified for neoadjuvant treatments in future clinic trials.

## Supplementary Material

zrad139_Supplementary_Data

## Data Availability

Data used in this manuscript are available from the corresponding author on reasonable request.
